# The patients’ perspective on the burden of idiopathic intracranial hypertension

**DOI:** 10.1186/s10194-021-01283-x

**Published:** 2021-07-08

**Authors:** Max Witry, Christine Kindler, Johannes Weller, Andreas Linder, Ullrich Wüllner

**Affiliations:** 1 Department of Neurology, University Hospital Bonn, Venusberg-Campus, 1, 53127 Bonn, Germany; 2 German Society of Intracranial Hypertension, Bad Honnef, Germany

**Keywords:** Pseudotumor cerebri, Obesity, Headache, Sleep disturbances, Depression

## Abstract

**Background:**

Idiopathic intracranial hypertension (IIH) is characterized by increased intracranial pressure without evidence of a tumor or any other underlying cause. Headache and visual disturbances are frequent complaints of IIH patients, but little is known about other symptoms. In this study, we evaluated the patients’ perspective on the burden of IIH.

**Methods:**

For this cross-sectional study, we developed an online survey for patients with IIH containing standardized evaluations of headache (HIT-6), sleep (PROMIS Sleep Disturbance Scale) and depression (MDI) in relation to BMI, lumbar puncture opening pressure (LP OP) and treatment.

**Results:**

Between December 2019 and February 2020, 306 patients completed the survey. 285 (93 %) were female, mean age was 36.6 years (± 10.8), mean BMI 34.2 (± 7.3) and mean LP OP at diagnosis was 37.8 cmH_2_O (± 9.5). 219 (72 %) of the participants were obese (BMI ≥ 30); 251 (82 %) reported severe impacting headaches, 140 (46 %) were suffering from sleep disturbances and 169 (56 %) from depression. Higher MDI scores correlated with higher BMI and increased sleep disturbances. Patients with a normalized LP opening pressure reported less headaches, less sleep disturbances and less depression than those with a constantly elevated opening pressure.

**Conclusions:**

In addition to headaches and visual disturbances, sleep disturbances and depression are frequent symptoms in IIH and contribute to the patients’ burden. Structured questionnaires can help to identify IIH patients’ needs and can lead to personalized and better treatment.

**Supplementary Information:**

The online version contains supplementary material available at 10.1186/s10194-021-01283-x.

## Introduction

Idiopathic intracranial hypertension (IIH) or pseudotumor cerebri is a neurological disorder characterized by increased intracranial pressure without evidence of a tumor or any other underlying disease [1–3]. In the 90 s, IIH was considered a rare condition with an initial incidence of one per 100.000 in the general population [4]. In line with the world-wide increase in obesity, the incidence of IIH raised considerably from 2.3 to 2003 to 7.8 per 100.000 in 2017 [5]. Although it can affect all subgroups of the population, studies show a significantly higher risk for young, overweight women of childbearing age, as well as people from lower socioeconomic backgrounds [5, 6]. Main symptoms are positional headaches, variable vision disturbances, pulsatile tinnitus and generalized weakness. The headache is usually bilateral and fronto-retroorbital, typically described as pressing or pulling, and occurs especially in the morning or during Valsava manoeuvres [7]. As the acronym implies, the cause of IIH remains unknown and treatment options are focused on reducing intracranial pressure to prevent visual loss [8–10]. While visual disturbances are reported in about 50 % of patients, complete loss of vision is thought to occur in 1–2 % of cases per year [11, 12]. CSF diversion through a lumbo- or ventriculoperitoneal shunt or optic nerve sheath fenestration are surgical options that may prevent irreversible blindness if carried out in a timely manner and are performed in approximately 9 % of IIH patients [13]. Visual disturbances and pain syndroms reduce the quality of life in IIH patients [14].To broaden the understanding of patients’ symptom burden, we aimed to investigate the role of sleep disturbances, depression and psychosocial aspects in IIH patients.

## Methods

### Survey design

For this cross sectional study, we developed an online survey for patients with IIH, which was carried out using LimeSurvey version 2.56.1. The first part of the questionnaire consisted of 20 open questions and was designed to explore the basic characteristics, including age, sex, size, weight, time since diagnosis as well as the lumbar puncture (LP) opening pressure at diagnosis (OPD) and most recently measured (OPR). We collected additional data on visual disturbances, number of LP, presence of post-lumbar puncture headache, current medication, and psychosocial aspects of the disease, such as the perception of LP, patient satisfaction and physician-patient communication (Additional file 1 in ESM). The second part evaluates the main topics of interest (headaches, sleep and mood disturbances), using validated and standardized questionnaires. For assessment of the impact of headache in daily life, the 6 items Headache Impact Test (HIT-6) is used, for sleep quality the Patient-Reported Outcomes Measurement Information System Sleep Disturbance Short form 8a (PROMIS-SD) and for depression the Major Depression Inventory (MDI) [15–17]. Exclusion criteria for further statistical analysis were incomplete data collection for the main parameters (BMI, LP OPD, HIT 6, MDI, PROMIS) or incoherent data (age < 18 or > 100 years, LP OPD < 20 cmH_2_O or > 60 cmH_2_O, number of lumbar punctures > 100).

### Patient recruitment

The German Society for Intracranial Hypertension invited patients diagnosed with IIH (self-report) on their social media platform (https://www.dgih.org/f) to participate in our survey via an encrypted link. In addition, IIH patients treated in our clinic were invited to participate in the study. All data were collected anonymously, and consent was obtained before participation in the survey. The Research Ethics Committee of the University Hospital in Bonn has confirmed that no ethical approval was required for this observational study.

### Data analysis

Standard descriptive measures are provided, including mean (± standard deviation) and frequency distribution where appropriate. Correlations were studied using Spearman’s rank order and correlation and multivariable logistic regression analyses were used to identify independent predictors for depression. Mann-Whitney rank sum test and Kruskal-Wallis test were employed for comparison of two or more than two groups, respectively. Post-hoc analyses were calculated with Dunn-Bonferroni-Test. Cohen’s classification was used to assess the effect size (r). An alpha level of 0.05 was considered statistically significant and all tests are two-sided. Data analysis was performed with SPSS version 25 (Armonk, NY: IBM Corp.).

## Results

Between December 2019 and February 2020, 527 of estimated 1000 invited active members of the German Society of Intracranial Hypertension participated in the survey. 159 (30 %) patients were excluded due to incomplete information and 62 (12 %) because of incoherent data as defined above. The mean time to complete the questionnaire was 13.5 min (± 7.6 min). Among the 306 participants available for analysis, 285 (93 %) were female and the mean age was 36.6 years (± 10.8, Table 1). The mean time since diagnosis was 4.2 years (± 4.0) and a mean time interval between symptom onset and diagnosis of 3.0 years (± 4.9 months) was stated. Upon diagnosis a mean LP OPD of 37.8 cmH_2_O (± 9.5) was reported. 219 (72 %) of the participants were obese; 114 (37 %) of them reported weight-gain shortly before diagnosis.

**Table 1:** Demographic and clinical characteristics of the participants

Characteristics	Mean ± SD	Min. - Max.
Age [years]	36.6 ± 10.8	18–61
BMI [kg/m^2^]	34.2 ± 7.3	15–59
Time since diagnose [years]	4.2 ± 4.0	1–21
LP OPD [cmH_2_O] ^a^	37.8 ± 9.5	20–60
LP OPR [cmH_2_O] ^b^	29.7 ± 9.2	10–57
HIT-6 ^c^	62.1 ± 5.4	36–66
PROMIS SD (T-score*) ^d^	58.8 ± 8.1	28.9–76.5
MDI^e^	26.7 ± 12.9	0–54

^a^Lumbar puncture opening pressure at diagnose (LP OPD)

^b^Lumbar puncture opening pressure, most recently measured (LP OPR)

^c^Headache Impact Test < 50: no impact, HIT-6 50–55: moderate impact, HIT-6 56–59: substantial impact and HIT-6 ≥ 60: severe impact

^d^PROMIS Sleep disturbance short form 8a T-score < 55 %: no, T-score 55–60: mild, T-score 60–70: moderate and T-score > 70: severe sleep disturbances, *raw score converted to T-score

^eMajor Depression Inventory 0–20: no depression, MDI 21–25: mild depression, MDI 26–30: moderate depression and MDI 31–50: severe depression^

In terms of treatment, 200 (65 %) reported taking carbonic anhydrase inhibitors: 114 (37 %) received acetazolamide, 31 (10 %) topiramate and 55 (18 %) reported taking both. In addition, the survey revealed that 101 (33 %) of the respondents were taking antidepressants, 79 (26 %) oral contraception and 28 (9 %) oral antidiabetics. Patients who took acetazolamide showed a trend towards better MDI scores than patients on topiramate or without medication (*p* = 0.246, supplementary Table 1 in ESM). Patients reported having received an average of 15 (± 13.1) lumbar punctures. 240 (78 %) patients found the LP extremely uncomfortable, 251 (82 %) reported post-lumbar puncture headaches.

**Fig. 1:**
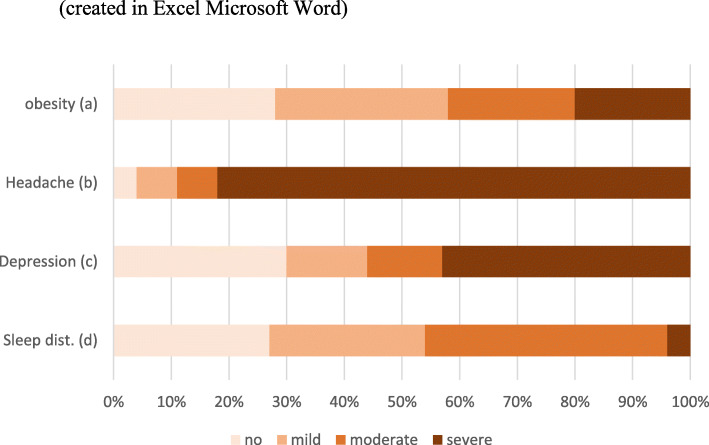
Patient distribution (%) of obesity (BMI), headache impact (HIT-6), depression (MDI) and sleep disturbances (PROMIS SD). **a.** BMI < 30: 28 %, BMI 30-34.9: 30 %, BMI 35–40: 22 % and BMI ≥40: 20 %. **b**. Headache Impact Test < 50: 4 %, HIT-6 50–55: 7 %, HIT-6 56–59: 7 % and HIT-6 ≥ 60: 82 %. **c**. Major Depression Inventory 0–20: 30 %, MDI 21–25: 14 %, MDI 26–30: 13 % and MDI 31–50: 43 %. **d**. PROMIS SD Short form 8a (T-score < 55): 27 %, T-score 55–60: 27 %, T-score 60–70: 42 % and T-score > 70: 4 %

The majority of the patients reported a high symptom burden in the standardized questionnaires. 251 (82 %) reported a severe impact of headaches on their daily life (HIT-6 ≥ 60), 140 (46 %) reported suffering from moderate or severe sleep disturbances (PROMIS SD T-score ≥ 60) and 169 (59 %) suffering from moderate or severe depression (MDI > 25, Fig. 1). 236 (77 %) reported frequent visual problems and 116 (49 %) of these felt that their daily life was very often severely impacted as a result. Furthermore, 184 of the patients (60 %) complained about a lack of psychological support and 245 (80 %) claimed that physicians were insufficiently informed about the disease.

**Fig. 2:**
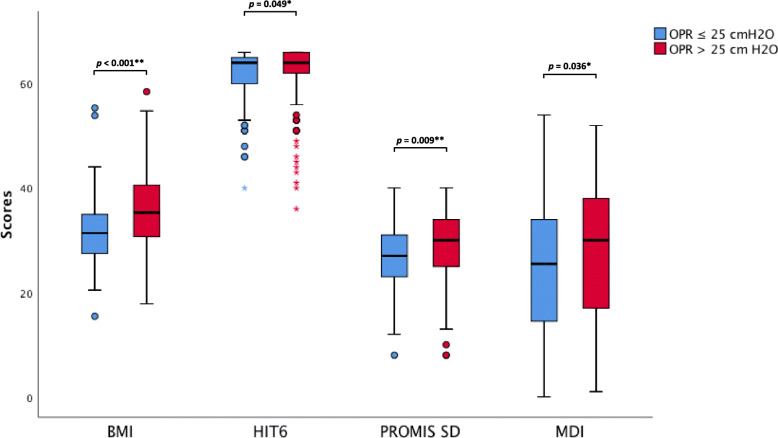
Comparison of LP OPR ≤ 25 cmH_2_O and LP OPR > 25 cmH_2_O regarding BMI, HIT-6, PROMIS SD and MDI *LP OPR.* most recently measured lumbar puncture opening pressure, *BMI* Body Mass Index (kg/m^2^), *HIT-6* Headache Impact Test, *PROMIS SD* Patient-Reported Outcomes Measurement Information System Sleep Disturbance Short form 8a, *MDI* Major Depression Inventory, * statistically significant (*p* < 0.05)

In Spearman’s correlation analysis, BMI (*r *= 0.2, *p* < 0.001), HIT-6 (*r* = 0.5, *p* < 0.001) and PROMIS SD score (*r* = 0.1, *p* < 0.001) were all correlated with MDI scores, while OPR was not (*p* = 0.124). In line with these findings, patients with depression (MDI > 20) were more frequently obese and reported both a stronger impact of headaches on their daily life and more severe sleep disturbances. With regard to sleep disturbances, post-hoc analyses showed a strong effect size between MDI ≤ 20 and MDI > 30 (*r* = 0.5, *p* < 0.001), a medium effect size between MDI ≤ 20 and MDI 21–30 (*r* = 0.3, *p* = 0.003) and a small effect size between MDI 21–30 and > 30 (*r* = 0.2, *p* = 0.006; supplementary Tables 2 and 3 in ESM). In multivariable logistic regression analysis adjusted for age and gender, the presence of obesity, sleep disturbances and severe impact of headaches on daily life were independent predictors for depression (MDI > 20; Table 2). Finally, patients with an OPR ≤ 25 cmH_2_O had a lower BMI (*r* = 0.3, *p* < 0.001) and reported lower scores in all questionnaires compared to patients with an OPR > 25 cmH_2_O: HIT-6 (*r* = 0.1, *p* = 0.049), PROMIS SD (*r* = 0.2, *p* = 0.009) and MDI (*r* = 0.1, *p* = 0.036; Fig. 2 and supplementary Table 4 in ESM).

**Table 2:** Multivariable logistic regression analysis of independent predictors of depression defined as MDI > 20, adjusted for age and sex

Item	aOR	95 % CI	Significance *p*
Obesity (BMI ≥ 30)	5.35	1.45–19.76	0.012*
Severe headache impact (HIT-6 ≥ 60)	2.06	1.19–3.58	0.010*
Relevant sleep disturbances (PROMIS SD T score ≥ 60)	4.36	2.50–7.61	< 0.001**

*BMI* Body Mass Index (kg/m^2^), *HIT-6* Headache Impact Test, *PROMIS SD* Patient-Reported Outcomes Measurement Information System Sleep Disturbance Short form 8a, *MDI* Major Depression Inventory, *aOR* adjusted odds ratio, *95 % CI* confidence interval *statistically significant (*p* < 0.05)

## Discussion

Headaches and visual disturbances are the most obvious symptoms in IIH and the main reason why IIH patients present to the neurologist or the ophthalmologist. In our study, more than 75 % of the participants reported visual disturbances and headaches severely impacting their daily lives. In addition to these cardinal symptoms of IIH, almost 50 % of the participants reported relevant sleep disturbances and depression. Since sleep disorders and depression are not described as being related to the disease, they might be insufficiently addressed in current clinical practice.

*Marcus et al.* considered sleep disturbances as a key risk factor for IIH and suggested that nocturnal hypercapnia is responsible for increased intracranial pressure and secondary papilledema [18]. Although obstructive sleep apnea syndrome (OSAS) is often described in IIH patients, it is not clear whether this is induced by IIH or due to the co-occurrence of obesity in IIH patients, which is a known risk factor for OSAS [19]. *Daniels et al.* showed a correlation between BMI and the risk of IIH [20], furthermore *Kesler at al.* demonstrated that increased weight is associated with recurrence of the disease [21]. In line with these findings, our survey revealed a strong interaction between a higher BMI and sleep disturbances. In addition, the study showed that in many cases, the onset of the disease was preceded by weight-gain.

Depression has an estimated lifetime prevalence of 15–20 % and severe depression has been identified in 37 % of IIH patients [22, 23]. An even higher rate of depression (56 %) was (self-) reported in our survey, with one third of the participants under an antidepressant medication. Important of note, topiramate (which 86 of the participants reported among their medication) may worsen depression and induce cognitive decline [24]. While the correlation of obesity and depression has been recognized earlier [25], depression also correlates with headaches and sleep disturbances. In our survey obesity, severe impact of headache in daily life and sleep disturbances were confirmed as independent predictors for depression.

60 % of the participants complained about a lack of information on IIH and 80 % claimed that physicians had insufficient knowledge about the disease. This suggests that physicians focus too much on LP OP and tend to perform procedures rather than consider psychological aspects. Repeated LP was perceived uncomfortable by many and post-lumbar puncture headaches may be understimated in IIH patients. Indeed, in a recent study by *Yiangou et al.*., the authors pointed out that LP should only be performed in severe headaches or to prevent visual loss [26]. After more invasive procedures like lumbo- or ventriculoperitoneal shunts, over half of the patients required shunt revision with the majority of these requiring multiple revisions. Therefore, CSF shunting should only be conducted as a last resort in otherwise untreatable, rapidly declining vision [27].

Depression and sleep disturbances can impair the ability to treat IIH and may particularly hamper weight loss. Weight gain and lack of exercise in turn promote the development of headache, sleep disturbances and depression. Patients thus find themselves in a vicious circle, with symptoms driving each other; an effect that may be exacerbated by the lockdown measures during the current pandemic [28]. We therefore strongly recommend the use of standardized questionnaires to assess patients’ symptoms, followed by multidisciplinary diagnosis and treatment, including referral to psychologists, psychiatrists and sleep physicians.

There are some limitations to our study that should be noted. First there was no external verification of the IIH diagnosis other than self-report and the same applies to all medical data, including symptoms and LP values. Second, the survey contained open questions, representing a subjective and individual view on the collected parameters. Third, there might be a selection bias, as patients with a higher level of suffering might be more likely to participate in the survey. Another limitation is that only 53 % of the invited population participated, leading primarily to a non-response bias. Possibly, IIH patients who are less severely or mildly affected tend to participate less in such surveys. A matched control population with similar gender, age and BMI would require the performance of a LP in healthy women, which is ethically questionable.

## Conclusions

In addition to headaches and visual disturbances, sleep disturbances and depression are frequent symptoms in IIH and contribute to the patients’ symptom burden. Clinicians should be aware that IIH patients may suffer from high levels of sleep disturbance and depression and assess their psychosocial needs, including their obvious need for more information and psychological support. Here we encourage the use of structured questionnaires, particularly MDI and PROMIS SD in patients with a BMI ≥ 30, to identify the affected patients and initiate further diagnostics and therapy where applicable. Future studies are needed to identify factors to improve the experience of the IIH patient.

## Supplementary Information

**Additional file 1: Supplementary Data.** Questionnaire. **Table 1.** Comparison of means (±SD) according to medication subgroups (Kruskal-Wallis-Test). **Table 2. **Comparison of means (±SD) according to MDI subgroups for BMI, headache (HIT-6), sleep disturbances (PROMIS SD) (Kruskal-Wallis-Test). **Table 3. **Posthoc Analysis comparing PROMIS scores (means of the raw score converted to T-score) within the different MDI subgroups (Dunn-Bonferroni Test). **Table 4. **Comparison of means (± SD) according to LP OPR for BMI, headache severity (HIT-6), sleep disturbances (PROMIS SD) and depression (MDI) (Mann-Whitney-U-Test).

## Data Availability

The Data can be obtained from the corresponding author upon reasonable request. Because of restrictions based on privacy regulations and informed consent of the participants, data cannot be made freely available in a public repository.

## Abbreviations

IIH: Idiopathic intracranial hypertension
HIT-6: 6 Items Headache Impact Test
PROMIS: Patient-Reported Outcomes Measurement Information System for Sleep disturbances
MDI: Major Depression Inventory
BMI: Body mass index
LP: Lumbar puncture
CSF: Cerebrospinal fluid
OPD: Opening pressure at diagnose
OPR: Opening pressure, most recently measured
OSAS: Obstructive sleep apnea syndrome
ESM: Electronic supplementary material

## References

[1] Friedman DI, Liu GT, Digre KB (2013). Revised diagnostic criteria for the pseudotumor cerebri syndrome in adults and children. Neurology.

[2] Mollan SP, Ali F, Hassan-Smith G (2016). Evolving evidence in adult idiopathic intracranial hypertension: pathophysiology and management. J Neurol Neurosurg Psychiatry.

[3] Zouari R, Messelmani M, Derbali H et al (2021) Idiopathic Intracranial Hypertension: an Unusual Presentation of Neuromyelitis Optica—A Case Report. SN Compr Clin Med 10.1007/s42399-021-00899-z

[4] Radhakrishnan K (1993). Idiopathic Intracranial Hypertension (Pseudotumor Cerebri): Descriptive Epidemiology in Rochester, Minn, 1976 to 1990. Arch Neurol.

[5] Miah L, Strafford H, Fonferko-Shadrach B (2021). Incidence, Prevalence and Healthcare Outcomes in Idiopathic Intracranial Hypertension: A Population Study. Neurology.

[6] Mollan SP, Aguiar M, Evison F (2019). The expanding burden of idiopathic intracranial hypertension. Eye.

[7] Peng MG, Gokoffski KK (2021). Updates on Recent Developments in Idiopathic Intracranial Hypertension. SN Compr Clin Med.

[8] Markey KA, Mollan SP, Jensen RH, Sinclair AJ (2016). Understanding idiopathic intracranial hypertension: mechanisms, management, and future directions. Lancet Neurol.

[9] Mollan SP, Hoffmann J, Sinclair AJ (2019). Advances in the understanding of headache in idiopathic intracranial hypertension. Curr Opin Neurol.

[10] Wall M, Kupersmith MJ, Kieburtz KD (2014). The idiopathic intracranial hypertension treatment trial: clinical profile at baseline. JAMA Neurol.

[11] Bouffard MA (2020). Fulminant Idiopathic Intracranial Hypertension. Curr Neurol Neurosci Rep.

[12] Best J, Silvestri G, Burton B, Foot B, Acheson J (2013). The Incidence of Blindness Due to Idiopathic Intracranial Hypertension in the UK. The Open Ophthalmology Journal.

[13] Thambisetty M, Lavin PJ, Newman NJ, Biousse V (2007). Fulminant idiopathic intracranial hypertension. Neurology.

[14] Digre KB, Bruce BB, McDermott MP (2015). Quality of life in idiopathic intracranial hypertension at diagnosis: IIH Treatment Trial results. Neurology.

[15] Kosinski M, Bayliss M, Bjorner J (2003). A six-item short-form survey for measuring headache impact: The HIT-6™. Qual Life Res.

[16] Yu L, Buysse DJ, Germain A (2011). Development of short forms from the PROMIS™ sleep disturbance and Sleep-Related Impairment item banks. Behav Sleep Med.

[17] Bech P, Rasmussen NA, Olsen LR (2001). The sensitivity and specificity of the Major Depression Inventory, using the Present State Examination as the index of diagnostic validity. J Affect Disord.

[18] Marcus DM, Lynn J, Miller JJ (2001). Sleep disorders: a risk factor for pseudotumor cerebri?. J Neuro-Ophthalmol.

[19] Thurtell MJ, Bruce BB, Rye DB (2011). The Berlin questionnaire screens for obstructive sleep apnea in idiopathic intracranial hypertension. J Neuro-Ophthalmol.

[20] Daniels AB, Liu GT, Volpe NJ (2007). Profiles of obesity, weight gain, and quality of life in idiopathic intracranial hypertension (pseudotumor cerebri). Am J Ophthalmol.

[21] Kesler A, Hadayer A, Goldhammer Y (2004). Idiopathic intracranial hypertension: risk of recurrences. Neurology.

[22] Kessler RC, Bromet EJ (2013). The epidemiology of depression across cultures. Annu Rev Public Health.

[23] Puustinen T, Tervonen J, Avellan C (2019). Psychiatric disorders are a common prognostic marker for worse outcome in patients with idiopathic intracranial hypertension. Clin Neurol Neurosurg.

[24] Mula M, Trimble MR, Lhatoo SD, Sander JWAS (2003). Topiramate and psychiatric adverse events in patients with epilepsy. Epilepsia.

[25] Mannan M, Mamun A, Doi S, Clavarino A (2016) Prospective Associations between Depression and Obesity for Adolescent Males and Females- A Systematic Review and Meta-Analysis of Longitudinal Studies. PLoS One 11(6):e0157240. doi 10.1371/journal.pone.0157240 10.1371/journal.pone.0157240 PMC4902254 27285386

[26] Yiangou A, Mitchell J, Markey KA (2019). Therapeutic lumbar puncture for headache in idiopathic intracranial hypertension: Minimal gain, is it worth the pain?. Cephalalgia.

[27] Sinclair AJ, Kuruvath S, Sen D (2011). Is cerebrospinal fluid shunting in idiopathic intracranial hypertension worthwhile? A 10-year review. Cephalalgia.

[28] Thaller M, Tsermoulas G, Sun R (2020). Negative impact of COVID-19 lockdown on papilloedema and idiopathic intracranial hypertension. J Neurol Neurosurg Psychiatry.

