# Supplementary material for: The patients’ perspective on the burden of idiopathic intracranial hypertension
Source: J Headache Pain. 2021 Jul 8;22(1):67. doi: 10.1186/s10194-021-01283-x (PMC8268504; doi:10.1186/s10194-021-01283-x)
Supplement: Supplementary file 1 — Additional file 1: Supplementary Data. Questionnaire. Table 1. Comparison of means (±SD) according to medication subgroups (Kruskal-Wallis-Test). Table 2. Comparison of means (±SD) according to MDI subgroups for BMI, headache (HIT-6), sleep disturbances (PROMIS SD) (Kruskal-Wallis-Test). Table 3. Posthoc Analysis comparing PROMIS scores (means of the raw score converted to T-score) within the different MDI subgroups (Dunn-Bonferroni Test). Table 4. Comparison of means (± SD) according to LP OPR for BMI, headache severity (HIT-6), sleep disturbances (PROMIS SD) and depression (MDI) (Mann-Whitney-U-Test). [file 10194_2021_1283_MOESM1_ESM.docx]

**Supplementary Data (Appendix 1):**

**Questionnaire**

**Basic characteristics:**

Date of birth: ____

Sex: male/female

Weight (kg): ______

Height (cm):_____

Did you gain weight shortly before diagnosis? yes/no

Have you lost weight since diagnosis? yes/no

**Medical history:**

Date of diagnosis: _____

Time interval between symptom onset and diagnosis: ____

Lumbar puncture opening pressure at diagnosis (LP OPD): ______

Most recently measured lumbar puncture opening pressure (LP OPR): ______

How many lumbar punctures have you had? _______

Have you experienced post-lumbar puncture headaches? yes/no

On a scale from 0 to 10 how uncomfortable would you rate a lumbar puncture? _____

Do you feel well informed about your disease? yes/no

Do you feel that your physician is well informed? yes/no

Do you lack psychological support? yes/no

**Visual disturbances:**

Have you ever had visual disturbances? yes/no

if yes: how often do these visual disturbances occur? never/rarely/sometimes/very often

if yes: how often are you restricted in your normal daily activities by the visual disturbances? never/rarely/sometimes/very often

**Medication:**

Have you taken any of the following medications for a prolonged period (>6 months)?

-Antidepressants (e.g. Sertraline, Citalopram, Venlaflaxine) yes/no, if so, which one? _____

-Antiepileptic drugs (e.g. Levetiracetam, Lamotrigine, Topiramate): yes/no, if so, which one? _____

-Diuretics (Acetazolamide or Glaupax, Furosemide): yes/no, if so, which one? _____

-Oral contraception? yes/no, if so, which one? _____

-Antidiabetics (e.g. Metformin, Januvia, Insulin): yes/no, if yes which one? _____

-any of the following (Cimetidine, Indometacin, Tamoxifen, Cyclosporine, Alpha interferon, Amiodarone, Tetracyclines, Nitrofurantoin, Nalidixic acid) if so which?_____

**Supplementary Data (Appendix 2):**

**Table 1: Comparison of means (±SD) according to medication subgroups (Kruskal-Wallis-Test).**

| Parameters | Medication | | | | Significance  p |
| --- | --- | --- | --- | --- | --- |
|  | TPM  (n=31) | AZM  (n=114) | TPM & AZM (n=55) | No medication  (n= 106) |  |
| BMI | 36.3 ± 8.3 | 32.8 ± 6.9 | 35.1 ± 7.3 | 34.6 ± 7.3 | 0.106 |
| HIT-6 | 63.2 ± 4.2 | 62.2 ± 4.8 | 61.8 ± 5.3 | 61.9 ± 6.2 | 0.670 |
| PROMIS SD | 29.1 ± 7.0 | 27.5 ± 7.0 | 28.8 ± 6.4 | 28.5 ± 7.0 | 0.492 |
| MDI | 28.9 ± 13.7 | 25.1 ± 13.1 | 26.2 ± 12.4 | 28.2 ± 12.5 | 0.246 |
| LP OPR | 31.1 ± 9.0 | 29.0 ± 9.0 | 30.6 ± 10.4 | 30.0 ± 8.6 | 0.639 |

*TPM* Topiramate, *AZM* Acetazolamide, *BMI* Body Mass Index (kg/m^2^), *HIT-6* Headache Impact Test, *PROMIS SD* Patient-Reported Outcomes Measurement Information System Sleep Disturbance Short form 8a, *MDI* Major Depression Inventory, *LP OPR* most recently measured lumbar puncture opening pressure

**Table 2: Comparison of means (±SD) according to MDI subgroups for BMI, headache (HIT-6), sleep disturbances (PROMIS SD) (Kruskal-Wallis-Test).**

| Parameters | MDI | | | Significance  p |
| --- | --- | --- | --- | --- |
|  | ≤20 (n=104) | 21-30 (n=70) | >30 (n=132) |  |
| BMI | 32.3 ± 7.0 | 33.8 ± 6.5 | 35.9 ± 7.6 | 0.001* |
| HIT-6 | 59.9 ± 6.4 | 62.2 ± 4.0 | 63.7 ± 4.4 | < 0.001** |
| PROMIS SD | 24.1 ± 7.3 | 28.4 ± 5.8 | 31.2 ± 5.4 | < 0.001** |

*MDI* Major Depression Inventory *, BMI* Body Mass Index (kg/m^2^), *HIT-6* Headache Impact Test, *PROMIS SD* Patient-Reported Outcomes Measurement Information System Sleep Disturbance Short form 8a, *statistically significant (p <0.05).

**Table 3: Posthoc Analysis comparing PROMIS scores (means of the raw score converted to T-score) within the different MDI subgroups (Dunn-Bonferroni Test).**

| MDI Subgroups | n | PROMIS | T-score | effect size r | Significance p |
| --- | --- | --- | --- | --- | --- |
| ≤20 vs. 21-30 | 174 | 24.1 vs. 28.4 | 54.3 vs. 58.3 | 0.3 | 0.003* |
| ≤20 vs. >30 | 235 | 24.1 vs. 31.2 | 54.3 vs. 61.3 | 0.5 | < 0.001** |
| 21-30 vs. >30 | 203 | 28.4 vs. 31.2 | 58.3 vs. 61.3 | 0.2 | 0.006* |

*MDI* Major Depression Inventory, *PROMIS SD* Patient-Reported Outcomes Measurement Information System Sleep Disturbance Short form 8 (raw score corresponding to the following T-score), effect size r (0.1-0.29): small, r (0.3-0.49): medium, r (≥0.5): strong effect, * statistically significant (p <0.05).

**Table 4: Comparison of means (± SD) according to LP OPR for BMI, headache severity (HIT-6), sleep disturbances (PROMIS SD) and depression (MDI) (Mann-Whitney-U-Test).**

| Parameters | LP OPR (cmH_2_O) | | Effect size  r | Significance  p |
| --- | --- | --- | --- | --- |
|  | ≤25 (n=110) | >25 (n=196) |  |  |
| BMI | 31.7 ± 6.1 | 35.6 ± 7.6 | 0,3 | < 0.001** |
| HIT-6 | 61.6 ± 5.3 | 62.4 ± 5.4 | 0,1 | 0.049* |
| PROMIS | 27.0 ± 6.5 | 28.9 ± 7.0 | 0,2 | 0.009* |
| MDI | 4.8 ± 12.3 | 27.8 ± 13.1 | 0,1 | 0.036* |

*LP OPR* most recently measured lumbar puncture opening pressure**,** *BMI* Body Mass Index (kg/m^2^), *HIT-6* Headache Impact Test, *PROMIS SD* Patient-Reported Outcomes Measurement Information System Sleep Disturbance Short form 8a, *MDI* Major Depression Inventory), effect size r (0.1-0.29): small, r (0.3-0.49): medium, r (≥0.5): strong effect, * statistically significant (p <0.05).
